# The CSF Levels of Neutrophil‐Related Chemokines in Patients with Neuromyelitis Optica

**DOI:** 10.1002/acn3.51094

**Published:** 2020-06-09

**Authors:** Zhuhe Liu, Jinyu Chen, Zhanhang Wang, Yao Wang, Dong Zheng, Honghao Wang, Yu Peng

**Affiliations:** ^1^ Department of Neurology Nanfang Hospital Southern Medical University Guangzhou China; ^2^ Department of Neurology Guangdong 999 Brain Hospital Guangzhou China; ^3^ Department of Neurology The Affiliated Brain Hospital of Guangzhou Medical University Guangzhou China

**Keywords:** neutrophil‐related chemokines, CXCL1, CXCL5, CXCL7, neuromyelitis optica

## Abstract

Pathologic findings showed that neutrophils played an important role in the pathogenesis of NMO. This study aims to investigate the CSF levels of neutrophil‐related chemokines in NMO. CXCL1, CXCL5, and CXCL7 were measured in 95 patients with NMO, 15 patients with MS, 18 patients with GFAP astrocytopathy, and 16 controls. The CSF level of CXCL1, CXCL5, and CXCL7 was significantly elevated in the NMO group but not correlated with the patient clinical severity. Besides, the CSF CXCL1, CXCL5, and CXCL7 could act as biomarkers to distinguish NMO from MS with good reliability, especially the CXCL7.

## Introduction

Neuromyelitis Optica (NMO) and multiple sclerosis (MS) are chronic disabling idiopathic inflammatory demyelinating diseases (IIDDs).^1^ Until Lennon *et al*. found the pathogenic anti‐NMO antibody (aquaporin‐4 antibody, AQP4) in 2004,^2^ NMO had long been considered as a specific variation of MS. Since then, more and more evidences have supported that they were two distinctive diseases.^3^ Pathological evidence has suggested that neutrophil infiltration in CNS lesions plays an important role in the pathogenesis of NMO,^4^ while the inflammatory leukocyte infiltrated in MS lesions are mainly macrophages and T lymphocytes.^5^, ^6^ Chemokines play an important role in the migration of leukocytes to CNS.^7^ CXCL chemokines (C‐X‐C motif chemokine ligand containing the glutamic acid, leucine, and arginine (ELR) motif) are the most important neutrophil‐related chemokine families,^8^ including CXCL1, CXCL5, CXCL7, CXCL10, and so on. The differences of these chemokines in NMO and MS patients may be an important factor which could account for the pathological differences. A few studies have reported the cerebrospinal fluid (CSF) levels of these chemokines in patients with MS ^9^, ^10^ and one study has reported the elevated plasma level of CXCL5 in NMO patients during remission,^11^ but little is known about the CSF levels of these neutrophil‐related chemokines in NMO patients during relapse. In the present study, we aimed to detect the levels of CXCL1, CXCL5, and CXCL7 in the CSF of patients with NMO and compared with patients with MS and autoimmune glial fibrillary acidic protein (GFAP) astrocytopathy.

## Methods and materials

### Patients and controls

Ninety‐five first diagnosis or relapsing NMO spectrum disorder (NMOSD) patients consistent with the 2015 Wingerchuk diagnostic criteria,^12^ 15 relapsing–remitting MS patients consistent with the 2017 McDonald’s diagnostic criteria,^13^ 18 GFAP astrocytopathy patients based on the 2018 diagnostic criteria,^14^ and 16 controls with non‐inflammatory neurological diseases (including cervical or lumbar spondylopathy, peripheral vertigo and so on) from the Department of Neurology, Nanfang Hospital, Southern Medical University were included. All patients signed the informed consent.

### Preparation of CSF samples

All patients were subjected to lumbar puncture for CSF analysis within 3 days after admission. CSF samples were centrifuged within 30 minutes. The supernatant was then transferred to polypropylene tubes and stored at −80°C until the assays were performed.

### Determination of CSF neutrophil‐related chemokines

Enzyme linked immunosorbent assay (ELISA) kits were purchased and used to quantify the CSF concentration of CXCL1, CXCL5, and CXCL7(R&D Systems ELISA Kits), according to the manufacturers’ instructions.

### Clinic evaluation

All NMOSD, MS, and GFAP patients were hospitalized during the first onset or relapses. The disease severity was assessed by Expanded Disability Status Scale (EDSS) scores. The clinical relapse of patients was defined as the onset of new symptoms that lasted at least 24 hours, and the EDSS score increased over 1.0 on admission.

### Statistical analysis

All statistical analyses were performed using SPSS version 20.0 (IBM Corp, Armonk, NY, USA). Data were expressed as mean (±standard deviation) or median (interquartile range). Kruskal–Wallis test was used for the comparison between groups. Correlations between EDSS and neutrophil‐related chemokines were evaluated using Spearman’s test. A value of *P* < 0.05 was considered statistically significant.

## Results

### Demographic and clinical features

The demographic data and clinical features of NMOSD patients (n = 95), MS patients (n = 15), GFAP patients (n = 18), and controls (n = 16) are shown in Table 1.

**Table 1:** The demographic data and clinical features of patients in each group

	NMO (n = 95)	MS (n = 15)	GFAP (n = 18)	CTLs (n = 16)
Gender (male/female)	13/82	9‐Jun	6‐Dec	11‐May
Age	43.9 ± 15.8	35.5 ± 11.6	41.7 ± 17.7	33.9 ± 2.9
Clinical symptoms				
Fever	7 (7%)	0 (0%)	14 (78%)	—
Dizziness	15 (16%)	2 (13%)	12 (67%)	—
Disorders of behavior or cognition	1 (1%)	2 (13%)	10 (56%)	—
Eye pain or Vision loss	30 (32%)	4 (26%)	1 (1%)	—
Abnormal feeling	53 (56%)	6 (40%)	2 (11%)	—
Autonomic disturbances	43 (45%)	1 (7%)	4 (22%)	—
Abnormal movements	1 (1%)	9 (60%)	12 (67%)	—
Epilepsy	6 (6%)	1 (7%)	2 (11%)	—
Vomit	17 (18%)	1 (7%)	4 (22%)	—
Lesion location				
Brain	6 (6%）	15 (100%)	14 (78%)	—
Spinal cord	74 (78%)	4 (27%)	4 (22%)	—
Brian and spinal cord	7 (7%)	4 (27%)	2 (11%)	—
Course (day, interquartile range)	12 (7, 30)	2 (3, 4.5)	10 (7, 15)	
EDSS (median, interquartile range)	3.0 (2.0, 6.5)	2.0 (2.0, 2.5)	3.5 (2.5, 4.5)	—
△EDSS (median, interquartile range)	2.0 (1.0, 3.5)	0.5 (0.5, 1.5)	3.0 (2.0, 4.0)	
CSF WBC (×10^6^/L, median (minimum–maximum))	0 (0‐420)	24 (0‐80)	24 (0‐110)	0 (0‐3)
CSF CXCL1 (pg/ml, mean ± SD)	18.8 ± 11.1	11.9 ± 3.0	12.1 ± 3.7	8.9 ± 1.5
CSF CXCL5 (ng/ml, mean ± SD)	41.6 ± 6.5	31.1 ± 13.9	34.2 ± 11.6	17.7 ± 5.3
CSF CXCL7 (ng/ml, mean ± SD)	466.7 ± 237.8	284.3 ± 155.2	347.6 ± 257.3	226.0 ± 33.1

CSF, Cerebrospinal Fluid; NMO, Neuromyelitis optica; MS, Multiple sclerosis; GFAP, glial fibrillary acidic protein; CTLs, controls; WBC, white blood cell; EDSS, Expanded Disability Status Scale; △EDSS, the change of EDSS after relapse.

### CSF CXCL1, CXCL5, and CXCL7 Levels

**Figure 1:**
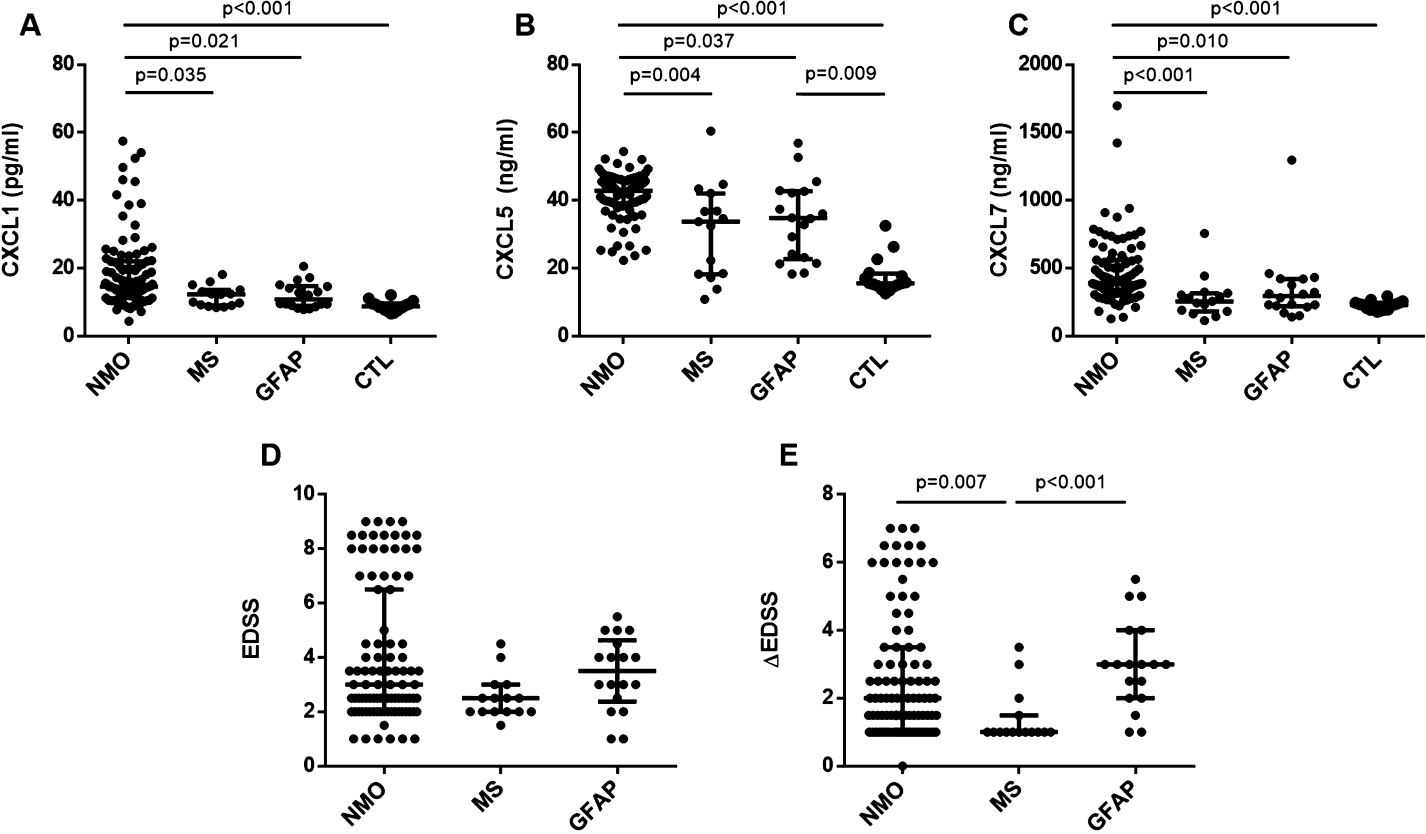
The CSF levels of CXCL1, CXCL5, and CXCL7 in NMOSD, MS, GFAP astrocytopathy patients and controls (A‐C). The EDSS and △EDSS scores in these three patients groups (D, E).

CSF concentration of CXCL1, CXCL5, and CXCL 7 was detected in patients and controls groups and the data were shown in Table 1 and Figure 1. Whether CXCL1, or CXCL5, or CXCL7, the concentration of these three chemokines in the NMO group were significantly higher than in the other three groups. The CSF concentration of CXCL5 in the GFAP group was significantly higher than in the controls. Meanwhile, these three chemokines showed no significant difference between the MS group and the GFAP group.

### Receiver operating characteristic (ROC) curve analysis

**Figure 2:**
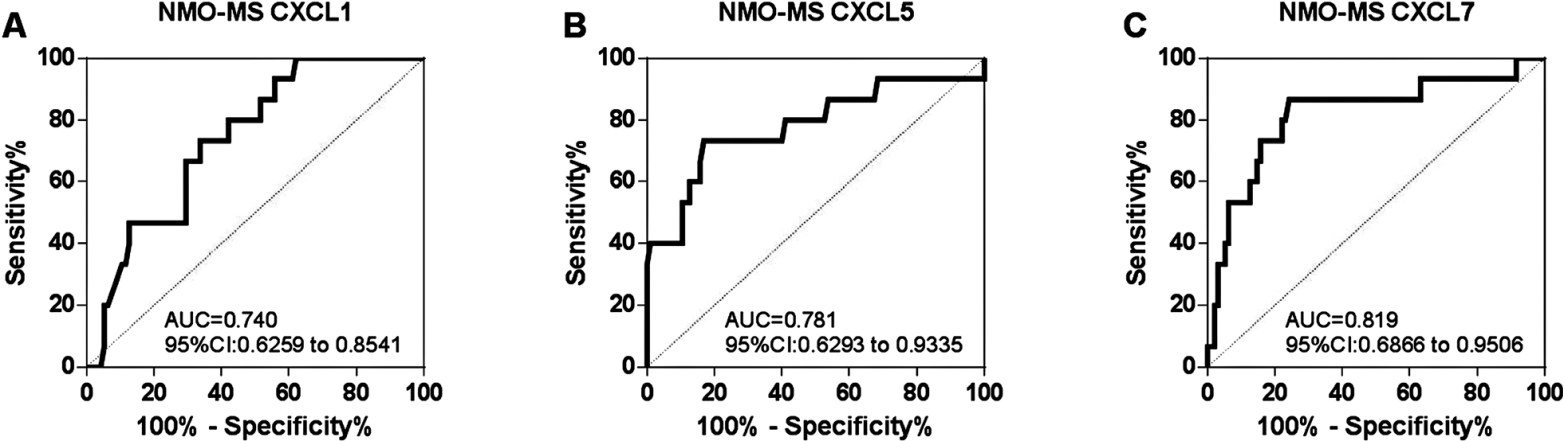
The ROC curve analysis of CSF CXCL1, CXCL5, and CXCL7 in NMOSD patients to distinguish from MS patients.

We performed ROC analysis for CXCL1, CXCL5, and CXCL7 to distinguish NMOSD patients from MS patients in Figure 2. The area under curve (AUC) for CXCL1 was 0.740 (95% CI: 0.626‐0.854, *P* = 0.003, best cut‐off value was 12.55 pg/ml with sensitivity 70.5% and specificity 66.7%). The AUC for CXCL5 was 0.781 (95% CI: 0.629‐0.934, *P < *0.001, best cut‐off value was 37.0 ng/ml with sensitivity 83.2% and specificity 73.3%). The AUC for CXCL7 was 0.819 (95% CI: 0.687‐0.951, *P < *0.0001, best cut‐off value was 325.3 ng/ml with sensitivity 75.8% and specificity 86.7%).

### Correlations between EDSS and neutrophil‐related chemokines

**Figure 3:**
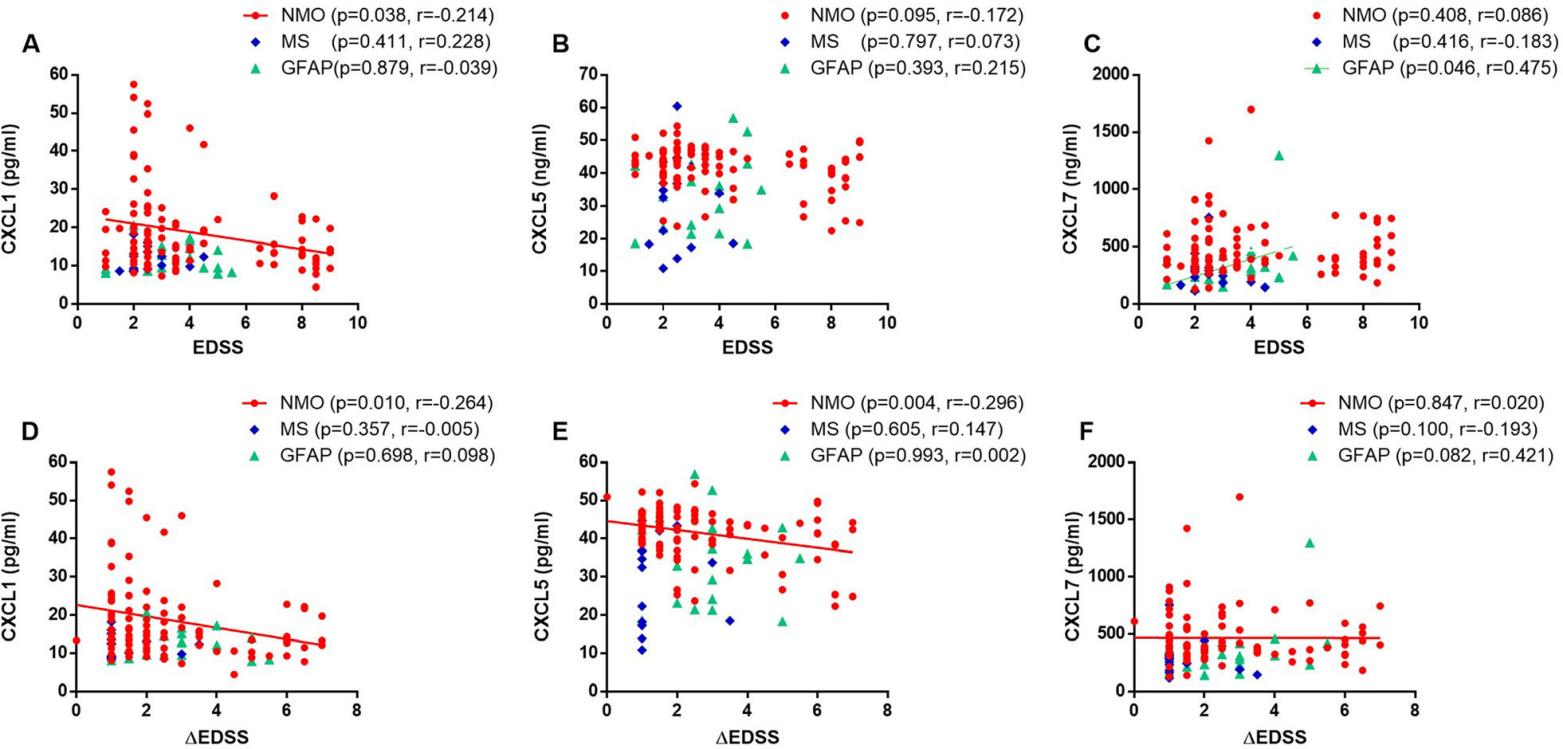
Correlation of CSF levels of CXCL1, CXCL5, and CXCL7 with the EDSS (A‐C) and △EDSS (D‐F) scores respectively in NMO, MS, and GFAP groups during relapse (Spearman’s test).

We analyzed the correlationship between EDSS, △EDSS (the difference of EDSS after and before relapse), and these three neutrophil‐related chemokines in each patients group, which is shown in Figure 3. CXCL1 had a significant negative correlation with EDSS (*r* = −0.214, *P* = 0.038) and △EDSS (*r* = −0.246, *P* = 0.010) only in NMO group. CXCL5 had a significant negative correlation with △EDSS (*r* = −0.296, *P* = 0.004) only in NMO group. CXCL7 had a significant positive correlation with EDSS (*r* = 0.475, *P* = 0.047) only in GFAP group.

## Discussion

In the present study, we measured the levels of CXCL1, CXCL5, and CXCL7 in the CSF of patients with NMOSD and compared with the patients with MS and GFAP astrocytopathy. All these three neutrophil‐related chemokines were significantly higher in the NMO. This result is consistent with the pathological findings that the CNS lesions of NMO were mainly infiltrated by neutrophils.^4^, ^15^

During the past two decades, more and more evidences had verified that NMO was a distinct disease from MS,^3^ especially in the pathological findings.^6^, ^16^ Autopsy had revealed that NMO patients had much more severe neutrophil infiltration in the CNS lesions than MS patients, and neutrophil was a very important factor for the inflammatory damage to CNS.^17^ It could account for why the clinical symptoms are more severe in NMO patients than in MS patients. It also suggested that neutrophils might play an important role in the pathogenesis of NMO. In recent years, more evidence showed that neutrophils displayed important functions for both innate and adaptive immune responses. Neutrophils can produce many cytokines and chemokines interacting with endothelial cells, dendritic cells, macrophages, NK cells, T cells, and B cells. We found CD40L and BAFF/APRIL systems were important for aggressive B cells and T‐cell responses, and might stimulate B‐cell activation in NMO.^18^, ^19^ Neutrophils could release the CD40L‐related molecules BAFF and APRIL which facilitated the survival of plasma cells emerging from antibody responses in NMO.^20^ Neutrophils activated the alternative complement pathway and released C5 fragments, which also further amplified neutrophil proinflammatory responses.^21^ An *in vitro* study showed that C5a‐preactivated neutrophils were critical for autoimmune‐induced astrocyte dysregulation in NMOSD.^22^ In an animal model of NMO, inhibiting neutrophil protease could reduce IgG induced brain damage.^23^ But in the present study, the CSF neutrophils count was not shown in the result, as the CSF WBC in most of NMO patients were normal (less than 8 × 10^6^/L) and it could not count how many neutrophils or lymphocytes were there.

How does neutrophils migrate to CNS from peripheral blood? Neutrophil‐related chemokines must play an important role.^7^ CXCL1, CXCL5, and CXCL7 are the members belong to the CXC chemokines family, which mainly help the migration of neutrophils through their common receptor CXCR2.^8^ Recently, studies on CXCL1, CXCL5, and CXCL7 mainly focus on the biomarker and pathogenesis of various malignant tumors,^24^, ^25^ but a few about CNS IIDDs existed. Rumble *et al*. reported elevated plasma CXCL5 in relapsing MS patients during acute lesion formation and the expression of CXCL1 and CXCL5 correlated with measures of MS lesion burden and clinical disability.^9^ Franciotta *et al*. reported that the CSF levels of CXCL1 and CXCL7 were similar as health controls in 14 MS patients.^10^ Yang et al. reported elevated plasma CXCL5 in NMO patients during remission.^11^ Little was known about these neutrophil‐related chemokines in the CSF of relapse NMO patients. As both NMO and MS are central nervous IIDDs, we thought that the neutrophil‐related chemokines released from central nervous system were more important for the central demyelinating lesions, and it might be reflected in the concentration of these chemokines in CSF.

As NMO and MS usually are very similar in clinic presentations, NMO used to be regarded as a variant of MS until the AQP4 antibody was discovered. But it was still sometimes difficult to distinguish these two diseases especially for the AQP4 antibody negative NMOSD patients.^26^ So, we hoped to find some biomarkers that can identify NMO and MS. In the present study, these CSF neutrophil‐related chemokines could act as diagnostic biomarkers for distinguishing NMO from MS, especially the CXCL7 had a good AUC value. In our previous studies, we had also detected some other valuable diagnostic biomarkers for NMO, such as neurofilament,^27^ soluble syndecan‐1 (sCD138),^28^ soluble cluster of differentiation 27 (sCD27),^29^ Chitinase‐3‐like‐1 (alias YKL‐40, yet not published), pyrin domain containing 3 (NLRP3) inflammasomes.^30^ These biomarkers represented neuron injury, endothelial injury, B lymphocyte activation, microglia activation, and general inflammation respectively, and indicated that the pathogenesis of NMO was multifactorial. So, it could account for why the EDSS or △EDSS do not have positive correlations with these neutrophil‐related chemokines.

GFAP astrocytopathy is a newly named disease in recent years, which is characterized by CSF positive anti‐GFAP antibody.^14^ However, it is still controversial whether GFAP antibody is the pathogenic antibody of this disease or secondary to the damage to astrocytes.^31^, ^32^ As some new evidences of pathology, NMOSD is also considered as an astrocytopathy.^33^ In the present study, the CSF levels of CXCL1, CXCL5, and CXCL7 in the GFAP group were significantly lower than those in the NMO group, suggested that the pathological mechanisms of the two diseases were different.

There were some limitations in the present study. Firstly, compared with the cases of NMO group, the sample size of MS group, GFAP group, and control group was a little small. It is due to the higher incidence of NMO than MS in Asian populations.^34^ Secondly, this is only a preliminary time/cross‐sectional designed study. Only 13 of 95 NMOSD patients received lumbar puncture and re‐evaluation of these neutrophil‐related chemokines during follow‐up. So it was difficult to evaluate the relationship between these chemokine changes and clinical recovery (data not shown). Finally, the exact effects of neutrophils in the pathogenesis of NMOSD are not clear and remains to be clarified.

## Conclusion

This study showed markedly higher levels of CSF CXCL1, CXCL5, and CXCL7 in the NMO. It could account for why NMO patients had much more severe neutrophil infiltration. It also suggested that neutrophils might play an important role in the pathogenesis of NMO. The CSF CXCL1, CXCL5, and CXCL7 could act as biomarkers to distinguish NMO from MS with good reliability, especially the CXCL7.

## Author Contributions

H. W., D. Z., and Y. P. conceived this study and designed the experiments. Z. L., J. C., Z. W., Y. W., and Y. P. collected the samples and clinical data. Z. L. and J. C. performed the experiments and analyzed the data. Y. P. wrote the manuscript and it was finally revised by D. Z. and H. W. All authors read and approved the final manuscript and agreed to submit it for publication.

## Conflict of Interest

All authors read and approved the final manuscript. They declare no conflicts of interest.

## Ethics Statement

The study protocol was approved by the Ethics Committee of Nanfang Hospital, Southern Medical University. Each participant provided written informed consent.

## References

[1] Pandit L , Asgari N , Apiwattanakul M , et al. Demographic and clinical features of neuromyelitis optica: a review. Mult Scler 2015;21(7):845–853. 25921037 10.1177/1352458515572406 PMC4463026

[2] Lennon VA , Wingerchuk DM , Kryzer TJ , et al. A serum autoantibody marker of neuromyelitis optica: distinction from multiple sclerosis. Lancet (London, England) 2004;364:2106–2112. 10.1016/S0140-6736(04)17551-X 15589308

[3] Argyriou AA , Makris N . Neuromyelitis optica: a distinct demyelinating disease of the central nervous system. Acta Neurol Scand 2008;118:209–217. 18336627 10.1111/j.1600-0404.2008.01002.x

[4] Lucchinetti CF , Mandler RN , McGavern D , et al. A role for humoral mechanisms in the pathogenesis of Devic's neuromyelitis optica. Brain 2002;125(Pt 7):1450–1461. 12076996 10.1093/brain/awf151 PMC5444467

[5] Kidd D , Barkhof F , McConnell R , et al. Cortical lesions in multiple sclerosis. Brain 1999;122(Pt 1):17–26. 10050891 10.1093/brain/122.1.17

[6] Kutzelnigg A , Lassmann H . Pathology of multiple sclerosis and related inflammatory demyelinating diseases. Handb Clin Neurol 2014;122:15–58. 24507512 10.1016/B978-0-444-52001-2.00002-9

[7] Scimone ML , Lutzky VP , Zittermann SI , et al. Migration of polymorphonuclear leucocytes is influenced by dendritic cells. Immunology 2005;114(3):375–385. 15720439 10.1111/j.1365-2567.2005.02104.x PMC1782099

[8] Griffith JW , Sokol CL , Luster AD . Chemokines and chemokine receptors: positioning cells for host defense and immunity. Annu Rev Immunol 2014;32:659–702. 24655300 10.1146/annurev-immunol-032713-120145

[9] Rumble JM , Huber AK , Krishnamoorthy G , et al. Neutrophil‐related factors as biomarkers in EAE and MS. J Exp Med 2015;212(1):23–35. 25559893 10.1084/jem.20141015 PMC4291533

[10] Franciotta D , Zardini E , Ravaglia S , et al. Cytokines and chemokines in cerebrospinal fluid and serum of adult patients with acute disseminated encephalomyelitis. J Neurol Sci 2006;247(2):202–207. 16784758 10.1016/j.jns.2006.05.049

[11] Yang T , Wang S , Zheng Q , et al. Increased plasma levels of epithelial neutrophil‐activating peptide 78/CXCL5 during the remission of Neuromyelitis optica. BMC Neurol 2016;16:96. 27401736 10.1186/s12883-016-0622-3 PMC4940958

[12] Wingerchuk DM , Banwell B , Bennett JL , et al. International consensus diagnostic criteria for neuromyelitis optica spectrum disorders. Neurology 2015;85(2):177–189. 26092914 10.1212/WNL.0000000000001729 PMC4515040

[13] Thompson AJ , Banwell BL , Barkhof F , et al. Diagnosis of multiple sclerosis: 2017 revisions of the McDonald criteria. Lancet Neurol 2018;17(2):162–173. 29275977 10.1016/S1474-4422(17)30470-2

[14] Fang B , McKeon A , Hinson SR , et al. Autoimmune glial fibrillary acidic protein astrocytopathy: a novel meningoencephalomyelitis. JAMA Neurol 2016;73(11):1297–1307. 27618707 10.1001/jamaneurol.2016.2549

[15] Khorooshi R , Asgari N , Mørch MT , et al. Hypersensitivity responses in the central nervous system. Front Immunol 2015;6:517. 26500654 10.3389/fimmu.2015.00517 PMC4595775

[16] Wegner C . Recent insights into the pathology of multiple sclerosis and neuromyelitis optica. Clin Neurol Neurosurg 2013;115(Suppl 1):S38–S41. 24321153 10.1016/j.clineuro.2013.09.019

[17] Pierson ER , Wagner CA , Goverman JM . The contribution of neutrophils to CNS autoimmunity. Clin Immunol 2018;189:23–28. 27377536 10.1016/j.clim.2016.06.017 PMC5203971

[18] Wang H , Wang K , Zhong X , et al. Cerebrospinal fluid BAFF and APRIL levels in neuromyelitis optica and multiple sclerosis patients during relapse. J Clin Immunol 2012;32(5):1007–1011. 22644841 10.1007/s10875-012-9709-9

[19] Zhong X , Wang H , Ye Z , et al. Serum concentration of CD40L is elevated in inflammatory demyelinating diseases. J Neuroimmunol 2016;15(299):66–69. 10.1016/j.jneuroim.2016.08.015 27725124

[20] Puga I , Cols M , Barra CM , et al. B cell‐helper neutrophils stimulate the diversification and production of immunoglobulin in the marginal zone of the spleen. Nat Immunol 2011;13(2):170–180. 22197976 10.1038/ni.2194 PMC3262910

[21] Camous L , Roumenina L , Bigot S , et al. Complement alternative pathway acts as a positive feedback amplification of neutrophil activation. Blood 2011;117(4):1340–1349. 21063021 10.1182/blood-2010-05-283564

[22] Piatek P , Domowicz M , Lewkowicz N , et al. C5a‐preactivated neutrophils are critical for autoimmune‐induced astrocyte dysregulation in neuromyelitis optica spectrum disorder. Front Immunol 2018;9:1694. 30083159 10.3389/fimmu.2018.01694 PMC6065055

[23] Saadoun S , Waters P , MacDonald C , et al. Neutrophil protease inhibition reduces neuromyelitis optica‐immunoglobulin G‐induced damage in mouse brain. Ann Neurol 2012;71(3):323–333. 22374891 10.1002/ana.22686 PMC3643520

[24] Strieter RM , Belperio JA , Phillips RJ , Keane MP . CXC chemokines in angiogenesis of cancer. Semin Cancer Biol 2004;14(3):195–200. 15246055 10.1016/j.semcancer.2003.10.006

[25] Guo Q , Jian Z , Jia B , Chang L . CXCL7 promotes proliferation and invasion of cholangiocarcinoma cells. Oncol Rep 2017;37(2):1114–1122. 27959418 10.3892/or.2016.5312

[26] de Seze J , Kremer L , Collongues N . Neuromyelitis optica spectrum disorder (NMOSD): a new concept. Rev Neurol (Paris) 2016;172:256–262. 27157418 10.1016/j.neurol.2016.03.003

[27] Wang H , Wang C , Qiu W , et al. Cerebrospinal fluid light and heavy neurofilaments in neuromyelitis optica. Neurochem Int 2013;63(8):805–808. 24161620 10.1016/j.neuint.2013.10.008

[28] Pei S , Zheng D , Wang Z , et al. Elevated soluble syndecan‐1 levels in neuromyelitis optica are associated with disease severity. Cytokine 2018;111:140–145. 30142535 10.1016/j.cyto.2018.08.017

[29] Liu B , Zhong X , Lu Z , et al. Cerebrospinal fluid level of soluble CD27 is associated with disease severity in neuromyelitis optica spectrum disorder. Neuroimmunomodulation 2018;25(4):185–192. 30423585 10.1159/000489561

[30] Peng Y , Chen J , Dai Y , et al. NLRP3 level in cerebrospinal fluid of patients with neuromyelitis optica spectrum disorders: Increased levels and association with disease severity [published online ahead of print. Mult Scler Relat Disord 2019;2019(39):101888. 10.1016/j.msard.2019.101888 31869599

[31] Shan F , Long Y , Qiu W . Autoimmune glial fibrillary acidic protein astrocytopathy: a review of the literature. Front Immunol. 2018;9:2802. 30568655 10.3389/fimmu.2018.02802 PMC6290896

[32] Zekeridou A , McKeon A , Flanagan EP . A path to understanding autoimmune GFAP astrocytopathy. Eur J Neurol 2018;25(3):421–422. 29193488 10.1111/ene.13527

[33] Lucchinetti CF , Guo Y , Popescu BF , et al. The pathology of an autoimmune astrocytopathy: lessons learned from neuromyelitis optica. Brain Pathol 2014;24(1):83–97. 24345222 10.1111/bpa.12099 PMC3905574

[34] Etemadifar M , Nasr Z , Khalili B , et al. Epidemiology of neuromyelitis optica in the world: a systematic review and meta‐analysis. Mult Scler Int 2015;2015:1–8. 10.1155/2015/174720 PMC4417948 25973275

